# Physician Beware: Severe Cyanide Toxicity from Amygdalin Tablets Ingestion

**DOI:** 10.1155/2017/4289527

**Published:** 2017-08-24

**Authors:** Tam Dang, Cham Nguyen, Phu N. Tran

**Affiliations:** ^1^Department of Emergency Medicine, Corona Regional Medical Center, Corona, CA, USA; ^2^Department of Pharmacy, University of California Irvine, Orange, CA, USA; ^3^Department of Hematology and Oncology, Genesis Cancer Center, Zanesville, OH, USA

## Abstract

Despite the risk of cyanide toxicity and lack of efficacy, amygdalin is still used as alternative cancer treatment. Due to the highly lethal nature of cyanide toxicity, many patients die before getting medical care. Herein, we describe the case of a 73-year-old female with metastatic pancreatic cancer who developed cyanide toxicity from taking amygdalin. Detailed history and physical examination prompted rapid clinical recognition and treatment with hydroxocobalamin, leading to resolution of her cyanide toxicity. Rapid clinical diagnosis and treatment of cyanide toxicity can rapidly improve patients' clinical outcome and survival. Inquiries for any forms of ingestion should be attempted in patients with clinical signs and symptoms suggestive of poisoning.

## 1. Introduction

Amygdalin (also known as Laetrile or vitamin B17) is a poisonous cyanogenic glycoside substance found naturally in many plants, including raw nuts such as bitter almonds and the pips of many fruits (particularly apricot pips or kernels). Amygdalin was isolated by French chemists in 1830 and it was first used as a treatment for cancer in Russia in 1845. In the United States, amygdalin was used in the 1920s. In the 1980s, The National Cancer Institute sponsored phase 1-2 clinical trials but found no evidence to support the clinical benefits of amygdalin in cancer treatment [1]. Furthermore, amygdalin was associated with cyanide poisoning, especially from oral ingestion [1]. As a result, the Food Drug Administration (FDA) banned the sale of amygdalin as a medicinal product. However, it remains available in the market and has been promoted as an alternative cancer treatment.

## 2. Case Report

A 73-year-old Arabic female with history of pancreatic adenocarcinoma came to the emergency department (ED) for altered level of consciousness via emergency medical service. History from the patient's son revealed that the patient was diagnosed with pancreatic cancer a few months ago and underwent Whipple surgery. Unfortunately, follow-up imaging revealed metastatic disease to her liver and lungs. She declined chemotherapy and recently began seeing a holistic medicine doctor who prescribed her vitamin B17 500 mg (amygdalin) tablets. On the day of presentation, she took three amygdalin tablets for the first time as instructed by her holistic medicine doctor. Approximately 45 minutes later, she developed altered mental status, along with diaphoresis, tachycardia, vomiting, dizziness, and crampy abdominal pain. In the emergency department, the patient was noted to be confused, making incomprehensible sound and withdrawing from pain with a GCS of 10. Her initial vital signs revealed a temperature of 37.2 degrees Celsius, heart rate of 133 beats per minute, respiratory rate of 13 acts per minute, blood pressure of 116/61 mm Hg, and an O2 saturation of 97% on 3 liters/minute nasal cannula. The patient had pale, cool, and red skin discoloration. We contacted the poison control center due to concern of cyanide toxicity from amygdalin ingestion, who recommended to give hydroxocobalamin (Cyanokit®) as an antidote if the patient had significant metabolic acidosis (pH < 7.2).

An hour into her ED stay, the patient's blood pressure decreased to 61/38 mm Hg. She was given 1 liter of normal saline, which improved her blood pressure to 92/48 mm Hg. Her laboratory data revealed a lactic acidosis of 14.2 mmol/L and an arterial blood gas (ABG) showed a pH of 7.24, pCO2 of 20 mmHg, a PaO2 of 141 mmHg, and HCO3^−^ of 8.4 mmol/L on 3 L/min nasal cannula. The patient's other abnormal laboratory tests included a leukocyte count of 27.9 × 10^3^ cells/*μ*L, HCO3^−^ of 11.0 mmol/L, anion gap of 23 mmol/L, glucose level of 255 mg/dL, estimated GFR of 44.80 mL/min/1.73 m2 according to the Chronic Kidney Disease Epidemiology Collaboration (CKD-EPI) equation. Her other electrolytes and liver function tests were within normal range. Her clinical deterioration led us to initiate 5 grams of intravenous hydroxocobalamin over 15 minutes. Approximately an hour after the administration of hydroxocobalamin, her blood pressure improved to 99/63 mmHg. At 2 hours after treatment, her blood pressure improved to 113/72 mm Hg and lactic acid level 6.5 mmol/L. Her mental status returned to baseline at 3 hours and a repeat ABG at that time revealed a pH of 7.32, pCO2 of 33 mmHg, a PaO2 of 129 mmHg, and HCO3^−^ of 16.8 mmol/L on 3 L/min nasal cannula. The patient was subsequently admitted to the ICU for close monitoring. Her clinical condition improved overnight in the ICU and she was discharged home the next day with instructions to stop B17 treatment permanently and follow up with her oncologist. Her repeat laboratory tests showed a leukocyte count of 12.2 × 10^3^ cells/*μ*L, HCO3^−^ of 18.0 mmol/L, anion gap of 8, glucose level of 98 mg/dL, and estimated GFR of 85.95 mL/min/1.73 m2 according to the CKD-EPI equation.

## 3. Discussion

Upon ingestion, amygdalin is hydrolyzed to cyanide by beta-glucuronidase in the small intestine [2]. Oral intake of 500 mg of amygdalin may contain as much as 30 mg of cyanide [3]. Oral amygdalin is estimated to be 40 times more potent than intravenous form due to its enzymatic conversion to hydrogen cyanide in the gastrointestinal tract [4]. Cyanide toxicity is highly fatal by interfering with mitochondrial oxygen utilization leading to cell death [5]. A minimum lethal dose of cyanide is approximately at 50 mg or 0.5 mg/kg body weight [4]. Cyanide binds to the ferric ion on cytochrome oxidase and abruptly halts the electron transport chain and oxidative metabolism, resulting in cellular hypoxia and lactic acidosis. Mild to moderate cases of cyanide toxicity consist of tachycardia, headache, confusion, nausea, and weakness. Severe cases may present with cyanosis, coma, convulsions, cardiac arrhythmias, cardiac arrest, and death [5]. Intake of amygdalin with foods containing beta-glucuronidase such as bean sprouts, peaches, celery, and carrots or concurrent intake with high doses of vitamin C has been shown to increase the conversion of amygdalin to cyanide in vitro [6].

This patient ingested 1500 mg of amygdalin (vitamin B17) as prescribed, which contains approximately 90 mg of cyanide. This amount is about 1.8 times higher than the minimum lethal dose of 50 mg of cyanide. Consequently, she presented with multiorgan failure including encephalopathy, lactic acidosis, and shock. Treatment of cyanide intoxication responded dramatically to administration of fluids and hydroxocobalamin. The rationale for using hydroxocobalamin as an antidote to cyanide poisoning is based on its high affinity to the cyanide ion. More specifically, the cobalt atom in the porphyrin-like ring of hydroxocobalamin has greater affinity for cyanide than cytochrome oxidase [7]. Preclinical studies demonstrate that hydroxocobalamin can readily penetrate into heavily cyanide loaded cells and complex cyanide to form cyanocobalamin (vitamin B12) [8]. In animal models, the administration of hydroxocobalamin in beagle dogs with cyanide toxicity greatly reduced mortality over placebo (21% versus 82%) [9]. Hydroxocobalamin is generally well-tolerated with side effects including dose-dependent erythema, headache, gastrointestinal distress, pruritus, dysphagia, and infusion-site reactions [10]. A pustular rash on the face and neck may rarely occur and may take several weeks to resolve. Hydroxocobalamin treatment is not associated with methemoglobinemia and vasodilation as seen in amyl nitrite/sodium nitrite or hypotension as in sodium thiosulfate [11]. Due to the favorable safety and efficacy profile, hydroxocobalamin has been proposed as an antidote for cyanide poisoning and Cyanokit (hydroxocobalamin) was approved by FDA in 2006 [12]. While hydroxocobalamin can be used as a monotherapy, there are animal studies and case reports that have shown potential synergistic effects when combined with sodium thiosulfate [13–15]. A case report in 2015 showed that a combination of hydroxocobalamin and sodium thiosulfate can have a positive effect on survival without long-term neurological and visual sequelae in cases of massive cyanide poisonings [16].

In summary, this case illustrates that rapid recognition of clinical signs of cyanide toxicity and aggressive treatments are critical to successful clinical outcome. Cyanide toxicity should be a differential diagnosis in high-anion-gap metabolic acidosis (lactic acidosis). Emergency physicians should frequently review patients' medications, including complementary and alternative drugs. Hydroxocobalamin monotherapy was demonstrated to be safe and effective in reversing cyanide poisoning in our case. Combination therapy may be more effective; however, further studies are required. Finally, this case also prompts discussion of the need to regulate the complementary and alternative medicine industry.
